# Latent profile analysis and influence factors of neurodevelopment in children aged 0–6 years in China

**DOI:** 10.3389/fpsyg.2026.1705627

**Published:** 2026-07-06

**Authors:** Ruili Li, Rui Zhang, Xiaoguo Zheng, Hui Wang, Yuqiao Guo, Congying Chen, Hongfang Yu, Li Bai, Qiuju Li, Jia Luo, Yuanyuan Guo, Xiaodan Li, Shengmei Zhang, Yan Zhang, Lei Gao

**Affiliations:** 1 Capital Center for Children’s Health, Capital Medical University, Capital Institute of Pediatrics, Beijing, China; 2 Department of Maternal, Child and Adolescent Health, School of Public Health, Tianjin Medical University, Tianjin, China; 3 Fengtai Women & Children’s Health Hospital, Beijing, China; 4 Fangshan Maternal & Child Health Hospital, Beijing, China; 5 Nanmofang Community Health Service Center in Chaoyang District, Beijing, China; 6 Beijing Shijingshan Maternal and Child Health Hospital, Beijing, China; 7 Beijing Daxing Maternal and Child Care Hospital, Beijing, China; 8 Beijing Chaoyang District Maternal and Child Health Care Hospital, Beijing, China; 9 The First Affiliated Hospital of Tsinghua University, Beijing, China; 10 Haidian District Maternal and Child Health Care Hospital, Beijing, China; 11 Beijing Pinggu District Maternal and Child Health Hospital, Beijing, China; 12 Huairou District Maternal and Child Health Hospital in Beijing, Beijing, China

**Keywords:** Chinese children, early-life environmental exposures, influencing factor, latent profile analysis, neuropsychological development

## Abstract

**Background:**

Early childhood neuropsychological development exhibits substantial heterogeneity, yet the identification of distinct developmental profiles and their associated risk factors remains limited. This study aimed to delineate latent profiles of neuropsychological development in children aged 0–6 years and to identify factors influencing membership in these profiles.

**Methods:**

A total of 2,297 children (1,193 boys and 1,104 girls) were assessed using the Chinese Developmental Scale for Children – Second Edition (CDSC-II). Latent profile analysis (LPA) was conducted based on age-standardized percentile scores across five developmental domains: gross motor, fine motor, language, adaptive behavior, and social behavior. A one-step regression mixture model, adjusting for child age and sex, was employed to examine associations between profile membership and perinatal and demographic factors.

**Results:**

The LPA identified two distinct profiles: an Ordinary Development Group (28.6%, *n* = 656) and an Excellent Development Group (71.4%, *n* = 1,641), with good class separation (entropy = 0.818). In the regression mixture model, low birth weight (OR = 2.09, 95% CI: 1.19–3.67), preterm birth (OR = 5.23, 95% CI: 3.14–8.71), maternal pregnancy complications (OR = 1.43, 95% CI: 1.15–1.78), and older child age (OR = 1.02, 95% CI: 1.007–1.027) were significantly associated with increased odds of belonging to the Ordinary Development Group. Conversely, female sex (vs. male) was associated with lower odds of membership in the Ordinary Development Group (OR = 0.67, 95% CI: 0.54–0.83). Parenting style (parental vs. non-parental care) and the timing of complementary food introduction were not independently associated with profile membership after adjustment (both *p* > 0.05).

**Conclusion:**

These findings indicate that specific maternal environmental exposures during pregnancy and the postnatal period, alongside child demographic characteristics, are significantly associated with neurodevelopmental outcomes in early childhood. The results underscore the importance of early developmental screening and targeted intervention for high-risk children to optimize long-term neurodevelopmental trajectories.

## 1 Introduction

Prenatal and early life represent sensitive developmental windows during which environmental exposures can exert lasting effects on health trajectories (Hahad and Al-Kindi, 2024). The Developmental Origins of Health and Disease (DOHaD) paradigm emphasizes that early environmental exposures may permanently alter the structure and function of organs and tissues, with lifelong ramifications (Silveira et al., 2007; Gluckman et al., 2016; Warembourg et al., 2019; Sly et al., 2021). Characterizing these early, potentially modifiable factors offers opportunities for primordial prevention to promote public health.

In recent years, there has been growing interest in the impact of early-life environmental exposures on children’s neurodevelopmental outcomes (Lipner et al., 2023). For example, Miguel et al. (2019) summarized that adverse early-life environments (from fetal period to childhood) are closely related to brain volume, microstructure, and connectivity, while positive early experiences may also buffer some adverse effects across generations. However, several gaps remain in the literature.

First, cultural differences are often overlooked in the assessment of children’s neuropsychological development (Byrd et al., 2008). Many neuropsychological assessments used in China are translated or adapted from foreign tools; there is an urgent need for locally developed measures that integrate cultural factors (Ng et al., 2023). The Developmental Scale for Children Aged 0–6 Years (WS/T 580–2017), also known as the Chinese Developmental Scale for Children – Second Edition (CDSC-II) or the Children Neuropsychological and Behavior Scale (CNBS), was developed by the Capital Institute of Pediatrics. (National Health Commission of the People’s Republic of China, 2017). It is one of the most widely used clinical developmental diagnostic tests in China, with nationwide standardization and good reliability and validity (Jin et al., 2014; Yang, 2023).

Second, previous research has paid insufficient attention to the heterogeneity of children’s neurodevelopment. According to classic developmental theory, development proceeds at varying rates across children and unevenly across different domains within the same child (Berk, 2022). Therefore, when assessing neuropsychological development, it is important to consider potential heterogeneity across ability domains. Latent profile analysis (LPA) is a person-centered modeling approach (Howard and Hoffman, 2018) that classifies individuals into subgroups based on their response patterns on observed variables. LPA maximizes between-group variance and minimizes within-group variance, and statistical indices can be used to evaluate classification accuracy (Miranda et al., 2021; Yamamoto et al., 2023). As such, LPA is well-suited to describe heterogeneity in child neurodevelopment (Momany et al., 2023; Strobel et al., 2020; Weller et al., 2020).

Third, prior studies have often ignored the measurement error associated with latent class assignments when examining associations between exposures and outcomes. Some studies have used traditional regression models based on most-likely class assignments derived from LPA (Frounfelker et al., 2022; Otieno et al., 2023; Samerei et al., 2021). However, this approach is suboptimal because latent class membership is estimated with uncertainty; treating it as observed introduces bias and typically underestimates the strength of associations (Bolck et al., 2004; Vermunt, 2010). Regression mixture models (RMMs) address this issue by estimating covariate effects and latent class membership simultaneously within a one-step framework, thereby properly accounting for classification uncertainty (Wang and Bi, 2017; Lamont et al., 2016; Asparouhov and Muthén, 2014).

Therefore, this study used the CDSC-II (CNBS), a locally developed neurodevelopmental assessment tool, to conduct latent profile analysis on the age-standardized percentile scores of five developmental domains (gross motor, fine motor, language, adaptive behavior, social behavior) in a sample of Chinese children aged 0–6 years. We then used a one-step regression mixture model to examine the associations between early-life environmental exposures and latent class membership.

We hypothesized that: (a) there are individual differences in children’s neuropsychological development, reflected in varying levels across domains; (b) child age and sex may be associated with class membership; and (c) specific early-life exposures (e.g., low birth weight, preterm birth, maternal pregnancy complications, feeding practices, parenting style) are associated with membership in a lower-functioning developmental profile.

## 2 Methods

### 2.1 Research object

The study’s dataset originates from cohort databases titled “Study on Developmental Behavior Monitoring and Appropriate Techniques for Intervention of Developmental Abnormalities in Children Aged 0–6 Years.” and “A study on self-assessment tools for psychological and behavioral development of 0–3 year old children”. Utilizing a multi-stage stratified random sampling technique, the study selected four districts in urban and four in suburban areas, comprising two urban and two suburban districts each. Within these districts, 1–2 medical and health service institutions were chosen as testing sites. The study analyzed test data collected from May 1, 2021, to August 30, 2023.

*Inclusion Criteria*: Children aged 0–6 years residing in the jurisdiction for more than half a year were included.

*Exclusion Criteria*: Children suffering from various acute and chronic diseases were excluded.

The study was conducted under The Code of Ethics of the World Medical Association (Declaration of Helsinki). We also obtained informed consent from the parents of participants before the experiment. The research protocol of this study was approved by the Medical Ethics Committee of Capital Institute of Paediatrics (KSSHERLLM 2017013; SHERLL2022051).

### 2.2 Neuropsychological development assessment tools

Children’s neuropsychological development was evaluated using the WS/T 580–2017 Developmental Behavior Assessment Scale for 0–6 Year-Old Children (CDSC-II) — also referred to as the Children Neuropsychological and Behavior Scale (CNBS) — and a standardized toolbox. This assessment covers five developmental domains: gross motor, fine motor, language, adaptive ability, and social behavior, along with the total development quotient. Developmental Age (DA) and Developmental Quotient (DQ) represent the child’s developmental level, with a DQ score below 70 indicating developmental delay, between 70 and 80 suggesting a developmental delay margin, and scores of 80 or above signifying normal development (Li et al., 2023).

### 2.3 Investigation of environmental exposures in early life

A custom questionnaire was employed for a face-to-face survey covering various aspects:

*Demographic Data*: Parental information including household registration, address, age, occupation, and monthly income per capita.

*Maternal Health*: Pregnancy history, diseases during pregnancy (e.g., anemia, hypertension), and maternal weight gain.

*Birth Status*: Delivery mode, gestational week, and newborn measurements (length, weight, head circumference).

*Disease Status at Birth*: Conditions like intrauterine asphyxia, birth distress, and hypoxic–ischemic encephalopathy.

*Neonatal Period*: Incidences of hyperbilirubinemia, neonatal pneumonia, sepsis, convulsions, and allergies.

*Infancy Feeding*: Primary caregivers, breastfeeding status, and introduction of complementary foods (binary yes/no indicator, assessing whether solid or semi-solid foods had been introduced at the time of assessment).

### 2.4 Introduction to latent profile analysis (LPA)

LPA is a modeling approach that identifies potential subpopulations within a population based on specific variables. The LPA assumes that people can, with varying degrees of probability, be typed into categories (subgroups) with different personal and/or environmental attributes of the configuration profile (Spurk et al., 2020). Its equation model can be expressed as:


$$\sigma_{i}^{2}=\sum_{k=1}^{k}\pi_{k}{\left(\mu_{\mathrm{ik}}-\mu_{i}\right)}^{2}+\sum_{k=1}^{k}\pi_{k}\sigma_{\mathrm{ik}}^{2}$$

Where μ_ik_ and σ_ik_ represent profile-specific (k) means and variances for variable i, and πk indicates profile density, or the proportion of N participants that belong to profile k. LPAs assume (a) samples drawn from a heterogeneous population produce data that are a mixture of K profile-specific distributions; (b) observed y indicator variables are distributed normally; and (c) the profile-specific mean vectors μ_k_ are the profile-specific (k) observed variable means. If both separate mean vectors (μ_k_) and separate covariance matrices (∑k) were freely estimated for all of the K latent profiles contained in the data, the number of estimated parameters would quickly increase as the number of observed variables increased. Therefore, two LPA model constraints are commonly imposed to model y variation with a minimum number of estimated parameters. First, the local independence assumption states that conditional on correct latent profile extraction or enumeration, all y are uncorrelated within each k latent profile, and all k-specific off-diagonal covariance matrix elements are zero. Second, the homogeneity assumption states that profile-specific covariance matrix elements along the main diagonal are constrained to equality across all k (i.e., ∑k = ∑, Y_k_ ~ N [μ_k_, ∑]) (Lubke and Neale, 2006; Vermunt and Magidson, 2009). Together, local independence and homogeneity assume that latent profile-specific (k) covariance matrices are diagonal and homogeneous, and that latent profiles differ only in their y-variable location (μ_k_), not their y-variable relationship form (∑) (Lubke and Neale, 2006).

### 2.5 Statistical methods

Latent profile analysis indicators. The LPA was conducted using five continuous indicator variables corresponding to the five developmental domains of the CDSC-II: (1) Gross Motor, (2) Fine Motor, (3) Language, (4) Adaptive Behavior, and (5) Social Behavior. For each domain, we used age-standardized percentile scores as the LPA indicators. These percentile scores were calculated according to the CDSC-II manual as: Percentile = (child’s Developmental Age (DA) score / same-age-group normative median DA) × 100. The CDSC-II provides age-specific normative data based on a nationally representative sample of Chinese children (WS/T 580–2017). Using percentile scores rather than raw scores minimizes age-related confounding, as the percentiles represent a child’s relative standing within their age cohort.

Data preprocessing and missing data. The five domain percentile scores were examined for distributional properties prior to analysis. Skewness and kurtosis statistics were within acceptable ranges (all |skewness| < 2.0, |kurtosis| < 7.0), supporting the use of maximum likelihood estimation without additional transformations. Missing data were handled using Full Information Maximum Likelihood (FIML) under the assumption of missing at random (MAR). FIML uses all available information in the dataset to estimate model parameters without listwise deletion, preserving statistical power and producing unbiased estimates under MAR. The overall missing data rate was low (<5% for any single variable).

Model selection. We fitted models with 1 through 5 latent classes. Model fit was evaluated using the Akaike Information Criterion (AIC), Bayesian Information Criterion (BIC), adjusted BIC (aBIC), entropy, the Lo–Mendell–Rubin (LMR) test, and the bootstrap likelihood ratio test (BLRT). The smallest class proportion was also considered to ensure interpretability.

Regression mixture model. To examine the association between early-life environmental exposures and latent class membership, we used a one-step regression mixture model (RMM). In this approach, covariates predicting class membership are included directly in the LPA model, and all parameters (class profiles, class proportions, and covariate effects on class membership) are estimated simultaneously. This one-step approach properly accounts for classification uncertainty because it does not require assigning individuals to discrete classes; instead, each individual contributes to the likelihood through their posterior class membership probabilities (Asparouhov and Muthén, 2014).

The following covariates were included as simultaneous predictors of class membership, selected *a priori* based on the Developmental Origins of Health and Disease (DOHaD) framework and prior literature: low birth weight (yes/no), preterm birth (yes/no), maternal history of pregnancy complications (yes/no), introduction of complementary foods (yes/no), parenting style (parental care vs. non-parental care), child sex (male/female), and child age in months (continuous). No stepwise variable selection was used; all covariates were entered together.

Software and estimation. All analyses were conducted using Mplus version 8.3 (Muthén & Muthén, 1998-2017). The LPA models were estimated using robust maximum likelihood (MLR) estimation, which provides standard errors and model fit statistics robust to non-normality and non-independence of observations. Missing data were handled using FIML as noted above. Model convergence was assessed by examining the iteration history and ensuring that all models achieved proper convergence (no negative residual variances, all parameter convergence criteria met). The key Mplus syntax for the final 2-class regression mixture model is provided in the Supplementary Appendix S1.

Quality control. Before the project was carried out, the test personnel were uniformly trained and all passed the assessment. Before carrying out the actual inspection, the Child Health Care Practitioners (CHCP) were taught in practice, and a consistency test was carried out on the CHCP. Only when the consistency test reached 0.9 could they obtain the qualification of the main examination. The data in this study were entered into the growth and development test system for children aged 0–6 years, and a new data set was established for analysis after logical error checking.

## 3 Results

### 3.1 Basic demographic characteristics of the subjects

A total of 2,297 children were included in the study, including 1,193 boys and 1,104 girls. The mean age of fathers was 32.29 ± 3.91 years, and that of mothers was 30.82 ± 2.92 years. Detailed information can be found in Table 1. The early environmental exposure of the subjects can be seen in Table 2.

**Table 1:** Sociodemographic characteristics of the sample (*N* = 2,297).

**Variables**	***N***	**%**
Gender		
Male	1,193	51.93
Female	1,104	48.07
Household registration		
Beijing	1,561	67.96
Non-Beijing	736	32.04
Ethnicity		
Han	2,244	97.69
Non-Han	53	2.31
Father’s age		
21.0–29.9 years	392	17.07
30.0–39.9 years	1774	77.23
≥40 years	131	5.70
Mother’s age		
21.0–29.9 years	850	37.00
30.0–39.9 years	1,403	61.80
≥40 years	44	1.20
Father’s occupational type		
Government or Organizational Manager	219	9.53
Professional/Technical Personnel	401	17.46
Administrative or Relevant Personnel	819	35.66
Social Production, Service, or Life Service Personnel	189	8.23
Agriculture, Forestry, Livestock, Fishing, or Auxiliary Personnel	162	7.05
Other Unclassified Occupations	507	22.07
Mother’s occupational type		
Government or Organizational Manager	197	8.58
Professional/Technical Personnel	360	15.67
Administrative or Relevant Personnel	821	35.74
Social Production, Service, or Life Service Personnel	170	7.40
Agriculture, Forestry, Livestock, Fishing, or Auxiliary Personnel	16	0.70
Other Unclassified Occupations	733	31.91
Father’s education level		
High School or Below	151	6.57
Associate’s Degree or Bachelor’s Degree	1940	84.46
Graduate Degree	206	8.97
Mother’s education level		
High School or Below	130	5.66
Associate’s Degree or Bachelor’s Degree	1971	85.81
Graduate Degree	196	8.53
Family *per capita* monthly income level		
< 3,000 yuan	5	0.22
3001–5,000 yuan	961	41.84
>5,000 yuan	1,331	57.94

**Table 2:** Early-life environmental exposures (*N* = 2,297).

**Variables**	*N*	%
Maternal anemia during pregnancy		
Yes	96	4.18
No	2,201	95.82
Maternal hypertension during pregnancy		
Yes	99	4.31
No	2,198	95.69
Gestational diabetes		
Yes	248	10.8
No	2049	89.2
Maternal hypothyroidism		
Yes	78	3.4
No	2,219	96.6
Maternal abnormal pregnancy history *		
Abnormal Pregnancy	150	6.53
Normal Pregnancy	2,147	93.47
Delivery method		
Natural Vaginal Delivery	1,390	60.51
Cesarean Section	860	37.44
Forceps or Vacuum Extraction	47	2.05
Premature birth		
< 28 weeks	7	0.3
28-32 weeks	33	1.44
33-37 weeks	308	13.41
≥ 38 weeks	1949	84.85
Birth weight		
< 1,500 g	37	1.61
1500-2499 g	105	4.57
2500-4000 g	2022	88.03
> 4,000 g	133	5.79
Birth length		
< 46 cm	87	3.79
46-52 cm	2,127	92.6
> 52 cm	83	3.61
Head circumference at birth		
< 32 cm	68	2.96
32–34 cm	1734	75.49
> 34 cm	495	21.55
Singleton or multiple births		
Singleton	2,255	98.17
Twins or Multiples	42	1.83
Oxygen deprivation during delivery		
Yes	20	0.87
No	2,277	99.13
Severe neonatal infection		
Yes	49	2.13
No	2,248	97.87
Hyperbilirubinemia		
Yes	90	3.92
No	2,207	96.08
History of allergies		
Yes	91	3.96
No	2,206	96.04
Feeding method		
Breastfeeding	1,224	53.29
Formula Feeding	567	24.68
Mixed Feeding	506	22.03
Introduction of solid food		
Yes	2,107	91.73
No	190	8.27
Primary caregiver		
Parents	1,086	47.28
Grandparents	1,192	51.89
Babysitters	19	0.83

*Abnormal pregnancy history includes previous miscarriage (first trimester), stillbirth (second trimester), or history of stillbirth during pregnancy or childbirth.

### 3.2 Latent profile analysis results

The study extracted 1–5 latent class models, and the fit indices are shown in Table 3. The AIC, BIC, and aBIC decreased monotonically with the increase in the number of classes. However, we selected the 2-class solution based on a balanced consideration of multiple criteria:

1. Statistical adequacy: The 2-class model demonstrated the highest entropy (0.805) among all fitted models, indicating the clearest class separation. While the 3-class model had a significant LMR test (*p* < 0.001), its entropy was slightly lower (0.797). The 4-class model showed a non-significant LMR test (*p* = 0.0245, which does not survive multiple comparison correction), and its entropy dropped further (0.755). The 5-class model had a non-significant LMR test (*p* = 0.299) and an unacceptably small smallest class size (4.18%, *n* ≈ 96), which would compromise statistical power and generalizability.
2. Parsimony and interpretability: The 2-class solution distinguished children with generally excellent development from those with ordinary development, which has clear clinical and public health relevance. The higher-order solutions (3-, 4-, and 5-class) did not reveal qualitatively distinct developmental *profiles* (i.e., domain-specific patterns); rather, they primarily subdivided the Ordinary Development Group by severity level, producing classes that differed quantitatively but not qualitatively in their domain patterns. Results for the 3- and 4-class solutions are provided in **Supplementary Table S1** and **Supplementary Figures S1–S3**.
3. Replicability: Given that our sample, while large, was drawn from specific geographic regions, we prioritized a more parsimonious solution that would be more likely to replicate in independent samples.

**Table 3:** Model fit indexes.

Model	AIC	BIC	aBIC	entropy	LMR	P	B_LRT	P	MMCN
1	4,392	4,449	4,417						
2	2077	2,169	2,118	0.805	−2186.065	<0.001	−2186.065	<0.001	28.57%
3	1726	1852	1782	0.797	−1022.896	<0.001	−1022.896	<0.001	18.86%
4	1,296	1,457	1,368	0.755	−841.306	0.0245	−841.306	<0.001	17.42%
5	1,222	1,418	1,310	0.758	−620.338	0.2989	−620.338	<0.001	4.18%

AIC, Akaike Information Criterion; BIC, Bayesian Information Criterion; aBIC, Adjusted Bayesian Information Criterion; BLRT, Bootstrap Likelihood Ratio Test; aLMR, Adjusted Lo–Mendell–Rubin Likelihood Ratio Test.

**Figure 1:**
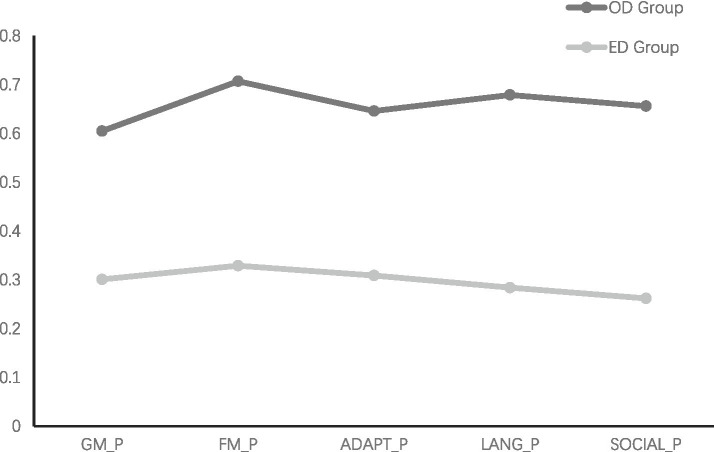
Percentile means for the five attributes within each latent class for Model 2/Two-class solution. OD Group = Ordinary Development Group (Class 1); ED Group = Excellent Development Group (Class 2); GM_P = Percentile of Gross Motor; FM_P = Percentile of Fine Motor, ADAPT_P = Percentile of Adaptive Behavior; LANG_P = Percentile of Language; SOCIAL_P = Percentile of Social Behavior.

Based on the mean percentile scores of the five developmental domains across classes (Figure 1), the boundary between the two classes was clear. Because Class 1 had lower mean scores across all five domains compared to Class 2, we named Class 1 “Ordinary Development Group” and Class 2 “Excellent Development Group.” In the whole sample, 656 children (28.56%) were classified into the Ordinary Development Group, and 1,641 children (71.44%) were classified into the Excellent Development Group. In the Ordinary Development Group, there were 379 boys and 277 girls; in the Excellent Development Group, there were 814 boys and 827 girls. There was a statistically significant difference in the sex ratio between the two groups (χ^2^ = 12.533, *p* < 0.001), with a higher proportion of girls in the Excellent Development Group. The average age of the Excellent Development Group was 12.933 ± 10.065 months, and the average age of the Ordinary Development Group was 14.964 ± 12.115 months; the difference was statistically significant (*t* = 4.173, *p* < 0.001). Results for the 3-class and 4-class solutions, including model fit indices and profile plots, are provided in Supplementary Table S2–S3 and Supplementary Figures S1–S2.

### 3.3 Factors associated with different profiles of neuropsychological development

According to the one-step regression mixture model adjusting for child age and sex, five early-life factors were significantly associated with latent class membership. Low birth weight (OR = 2.09, 95% CI: 1.19–3.67), preterm birth (OR = 5.23, 95% CI: 3.14–8.71), maternal pregnancy complications (OR = 1.43, 95% CI: 1.15–1.78), and older child age (OR = 1.02, 95% CI: 1.007–1.027) were associated with higher odds of belonging to the Ordinary Development Group. Female sex (vs. male) was associated with lower odds of belonging to the Ordinary Development Group (OR = 0.67, 95% CI: 0.54–0.83). Parental care (parental vs. non-parental care) and introduction of complementary foods were not independently associated after adjustment (both *p* > 0.05). Detailed results are presented in Table 4.

**Table 4:** Early environmental factors associated with membership in the Ordinary Development Group (vs. Excellent Development Group) (*N* = 2,297).

Variable	*B*	S.E.	*P*	OR	95% CI
Low birth weight (yes vs. no)	0.738	0.286	0.010	2.09	[1.19, 3.67]
Preterm birth (yes vs. no)	1.654	0.260	<0.001	5.23	[3.14, 8.71]
Maternal pregnancy complications (yes vs. no)	0.355	0.112	0.002	1.43	[1.15, 1.78]
Introduction of complementary foods (yes vs. no)	−0.111	0.118	0.348	0.90	[0.71, 1.13]
Parental care (vs. non-parental care)	−0.457	0.262	0.081	0.63	[0.38, 1.06]
Child age (months, continuous)	0.017	0.005	0.001	1.02	[1.007, 1.027]
Sex (female vs. male)	−0.407	0.111	<0.001	0.67	[0.54, 0.83]

*In this study, the mother’s history of abnormal pregnancy was a previous pregnancy, a miscarriage in the first trimester, a stillbirth in the second trimester, or a history of stillbirth during pregnancy or even childbirth. OR > 1 indicates higher odds of being in the Ordinary Development Group. All covariates were entered simultaneously. The model adjusted for child age and sex. B = log odds ratio; S.E. = standard error; CI = confidence interval.

## 4 Discussion

In recent years, both domestic and international scholars have increasingly adopted person-centered latent variable models to explore heterogeneity in child psychological development (Baltes et al., 2007; Beebe et al., 2016). Notably, research utilizing the Bayley Scales of Infant and Toddler Development to analyze very preterm infants has identified four latent neurobehavioral development profiles, a methodology paralleling our study (Camerota et al., 2024). Additionally, Dong et al. have delineated high-level and low-level development groups in their exploration of committed compliance developmental trajectories in typically developing infants and toddlers, employing latent class analysis (Dong et al., 2018).

Our investigation using the five age-standardized percentile scores of the CDSC-II revealed a two-class solution that primarily reflects a quantitative differentiation in overall developmental level rather than qualitatively distinct neurodevelopmental profiles. Specifically, children in the Excellent Development Group (Class 2) scored higher across all five domains (gross motor, fine motor, language, adaptive behavior, social behavior) in a relatively parallel fashion, while children in the Ordinary Development Group (Class 1) showed uniformly lower scores. This pattern is consistent with a general neurodevelopmental factor or g-factor commonly observed in developmental research (Tucker-Drob and Briley, 2014), where individual differences in development tend to be correlated across domains. Consequently, while latent profile analysis provides a useful empirical classification of children into higher- and lower-functioning groups, our findings do not support the existence of distinct, domain-specific developmental phenotypes in this sample. Future research with larger and more diverse samples may be needed to identify whether such phenotypes exist in the broader population of Chinese children.

This study indicates significant differences in gender and age within the two latent profiles, with girls more frequently classified into the Excellent Development Group, where members were also younger on average compared to the Ordinary Development Group. Historical research on gender’s impact on child psychological development suggests preschool-age girls possess advantages over boys in motor skills, language, and social development (Else-Quest et al., 2006; Clements et al., 2006; Dong et al., 2018; Matarma et al., 2020). One proposed rationale involves differences in brain hemisphere development, affording girls inherent advantages in brain functional organization, notably in language and communication (Adani and Cepanec, 2019). However, the argument that gender differences in brain structure or function directly translate to language task performance disparities remains contested, with limited evidence supporting gender differences in brain and language development (Etchell et al., 2018). This study’s observations of gender differences in neurodevelopment require further substantiation through additional methods, such as cognitive neurotests sensitive to age or neuroimaging studies.

Internationally, the consensus is that a child’s cognitive neurodevelopment level correlates strongly with age, reflected in age-specific developmental milestones (Misirliyan et al., 2023). Although we used age-standardized percentile scores to minimize the effect of age, the two latent classes still differed significantly in mean age, with the Ordinary Development Group being older on average (14.96 ± 12.12 months) than the Excellent Development Group (12.93 ± 10.07 months). This residual age difference may reflect the fact that older children in our sample had a broader range of developmental experiences and environmental exposures that could influence their relative standing on age-standardized percentiles. As a sensitivity analysis, we included child age (in months) as a continuous covariate in the regression mixture model; the pattern of associations remained substantively unchanged (see Supplementary Table S4), suggesting that the reported ORs are not primarily driven by age confounding. Nevertheless, residual age effects cannot be completely ruled out.

Using a one-step regression mixture model (RMM) that properly accounts for classification uncertainty, our study confirms associations (instead of causal effects) between several early-life environmental exposures and neurodevelopmental class membership. Children exposed to low birth weight (OR = 2.09, 95% CI: 1.19–3.67), preterm birth (OR = 5.23, 95% CI: 3.14–8.71), or maternal pregnancy complications (OR = 1.43, 95% CI: 1.15–1.78) were more likely to belong to the Ordinary Development Group. These findings align with the bulk of similar research (Allotey et al., 2018; Kyriakidou et al., 2020). Adverse maternal pregnancy histories may relate to a range of factors, including genetic inheritance, nutritional factors, and occupational hazards during pregnancy, potentially limiting offspring psychological development (Corchero-Falcón et al., 2023; Honigberg et al., 2023; Lipner et al., 2023; Malacova et al., 2018; Zhang et al., 2022). Further research is needed to elucidate the mechanisms through which maternal pregnancy history impacts offspring neurodevelopment.

In contrast, after adjustment for other covariates, introduction of complementary foods (OR = 0.90, 95% CI: 0.71–1.13, *p* = 0.348) and parental care (parental vs. non-parental caregiving; OR = 0.63, 95% CI: 0.38–1.06, *p* = 0.081) were not significantly associated with class membership. Although the direction of the point estimates is consistent with a potential protective effect, the lack of statistical significance suggests that these factors may not be independent contributors in this sample, or that their effects are smaller and require larger studies to be detected. Previous literature has emphasized the importance of early feeding practices and responsive caregiving (WHO, 2012; Boswell, 2021; English et al., 2019; Wang et al, 2024; Wei, 2019), but our null findings may reflect residual confounding by unmeasured socioeconomic or caregiving factors, or the relatively high baseline levels of complementary feeding (91.7% of children had been introduced) limiting variability.

Nevertheless, this study has several important limitations. First, the cross-sectional design precludes establishing temporal order between exposures and developmental outcomes. While many of the exposures (e.g., low birth weight, preterm birth, maternal pregnancy history) temporally precede the developmental assessment, other variables — notably parenting style and complementary food introduction — were measured concurrently with the outcome. Reverse causality is therefore a plausible alternative explanation: children with observed or perceived developmental delays may elicit different caregiving responses (e.g., more intensive parental care, altered feeding practices) or may be more likely to remain in parental rather than non-parental care. The direction of association cannot be determined from these data.

Second, several key exposures were assessed via caregiver self-report, which is subject to recall bias and social desirability bias. Maternal pregnancy history and feeding practices may be particularly susceptible to inaccurate recall, especially in a sample with children aged 0–6 years.

Third, the absence of detailed socioeconomic data (parental education, household income, maternal mental health) limits our ability to control for confounding by socioeconomic status, which is strongly associated with both the exposures and child developmental outcomes.

Fourth, although we used age-standardized percentile scores and additionally adjusted for age in sensitivity analyses, the significant age difference between the two latent classes suggests that residual age confounding cannot be entirely ruled out.

Fifth, as noted above, the two-class solution identified represents a general developmental level gradient rather than qualitatively distinct phenotypes. Whether this reflects a true absence of domain-specific profiles in this population or limited statistical power to detect such profiles deserves further investigation in larger samples.

## 5 Conclusion

This study used latent profile analysis to identify two quantitative developmental groups (Excellent and Ordinary) among Chinese children aged 0–6 years based on age-standardized CDSC-II percentile scores. The two-class solution primarily reflects a general developmental level gradient rather than qualitatively distinct domain-specific profiles. A one-step regression mixture model revealed that low birth weight, preterm birth, maternal pregnancy complications, older child age, and male sex are significantly associated with membership in the Ordinary Development Group (Class 1). Parenting style and introduction of complementary foods were not independently associated after adjustment. These findings highlight the importance of early-life environmental exposures for child neurodevelopment. However, due to the cross-sectional design, causal inferences cannot be drawn. Future longitudinal studies are needed to clarify the temporal direction of these associations and to explore whether more nuanced developmental phenotypes emerge in larger, more diverse samples.

## Data Availability

The raw data supporting the conclusions of this article will be made available by the authors, without undue reservation.

## References

[ref1] Adani S. Cepanec M. (2019). Sex differences in early communication development: behavioral and neurobiological indicators of more vulnerable communication system development in boys. Croat. Med. J. 60, 141–149. doi: 10.3325/cmj.2019.60.141, 31044585 PMC6509633

[ref2] Allotey J. Zamora J. Cheong-See F. Kalidindi M. Arroyo-Manzano D. Asztalos E. . (2018). Cognitive, motor, behavioural and academic performances of children born preterm: a meta-analysis and systematic review involving 64 061 children. BJOG 125, 16–25. doi: 10.1111/1471-0528.14832, 29024294

[ref3] Asparouhov T. Muthén B. (2014). Auxiliary variables in mixture modeling: three-step approaches using Mplus. Struct. Equ. Model. 21, 329–341. doi: 10.1080/10705511.2014.915181

[ref4] Baltes P. B. Lindenberger U. Staudinger U. M. (2007). “Life span theory in Developmental Psychology,” in Handbook of Child Psychology, (Hoboken, NJ: Wiley Press).

[ref5] Beebe B. Messinger D. Bahrick L. E. Margolis A. Buck K. A. Chen H. (2016). A systems view of mother-infant face-to-face communication. Dev. Psychol. 52, 556–571. doi: 10.1037/a0040085, 26882118 PMC4808406

[ref6] Berk L. E. (2022). Development Through the Lifespan. 7th Edn. Thousand Oaks, CA: SAGE.

[ref7] Bolck A. Croon M. Hagenaars J. (2004). Estimating latent structure models with categorical variables: one-step versus three-step estimators. Polit. Anal. 12, 3–27. doi: 10.1093/pan/mph001

[ref8] Boswell N. (2021). Complementary feeding methods-a review of the benefits and risks. Int. J. Environ. Res. Public Health 18:7165. doi: 10.3390/ijerph18137165, 34281101 PMC8297117

[ref9] Byrd D. Arentoft A. Scheiner D. Westerveld M. Baron I. S. (2008). State of multicultural neuropsychological assessment in children: current research issues. Neuropsychol. Rev. 18, 214–222. doi: 10.1007/s11065-008-9065-y, 18815888

[ref10] Camerota M. McGowan E. C. Aschner J. Stroustrup A. O'Shea T. M. Hofheimer J. A. . (2024). Neurodevelopmental and behavioral outcomes of very preterm infants: latent profile analysis in the environmental influences on child health outcomes (ECHO) program. Pediatr. Res. 95, 377–385. doi: 10.1038/s41390-023-02814-9, 37700161 PMC10885008

[ref11] Clements A. M. Rimrodt S. L. Abel J. R. Blankner J. G. Mostofsky S. H. Pekar J. J. . (2006). Sex differences in cerebral laterality of language and visuospatial processing. Brain Lang. 98, 150–158. doi: 10.1016/j.bandl.2006.04.007, 16716389

[ref12] Corchero-Falcón M. D. R. Gómez-Salgado J. García-Iglesias J. J. Camacho-Vega J. C. Fagundo-Rivera J. Carrasco-González A. M. (2023). Risk factors for working pregnant women and potential adverse consequences of exposure: a systematic review. Int. J. Public Health 68:1605655. doi: 10.3389/ijph.2023.1605655, 36874222 PMC9977819

[ref13] Dong S. Wang Z. Lu S. Liang X. Xing X. P. (2018). Children’s temperament and maternal behavioral control: origins of heterogeneity in developmental trajectories of committed compliance from infancy to age 3. J. Child Fam. Stud. 27, 2668–2677. doi: 10.1007/s10826-018-1101-9

[ref14] Else-Quest N. M. Hyde J. S. Goldsmith H. H. Van Hulle C. A. (2006). Gender differences in temperament: a meta-analysis. Psychol. Bull. 132, 33–72. doi: 10.1037/0033-2909.132.1.33, 16435957

[ref15] English L. K. Obbagy J. E. Wong Y. P. Butte N. F. Dewey K. G. Fox M. K. . (2019). Types and amounts of complementary foods and beverages consumed and growth, size, and body composition: a systematic review. Am. J. Clin. Nutr. 109, 956S–977S. doi: 10.1093/ajcn/nqy281, 30982866

[ref16] Etchell A. Adhikari A. Weinberg L. S. Choo A. L. Garnett E. O. Chow H. M. . (2018). A systematic literature review of sex differences in childhood language and brain development. Neuropsychologia 114, 19–31. doi: 10.1016/j.neuropsychologia.2018.04.011, 29654881 PMC5988993

[ref17] Frounfelker R. L. Li Z. Y. Santavicca T. Miconi D. Rousseau C. (2022). Latent class analysis of COVID-19 experiences, social distancing, and mental health. Am. J. Orthopsychiatry 92, 121–132. doi: 10.1037/ort0000593, 34914415

[ref18] Gluckman P. D. Buklijas T. Hanson M. A. (2016). “Chapter 1 - the developmental origins of health and disease (DOHaD) concept: past, present, and future,” in The Epigenome and Developmental Origins of Health and Disease, ed. Rosenfeld C. S. (Academic Press).

[ref19] Hahad O. Al-Kindi S. (2024). The prenatal and early life Exposome: shaping health across the lifespan. JACC Adv 3:100807. doi: 10.1016/j.jacadv.2023.100807, 38939401 PMC11198030

[ref20] Honigberg M. C. Truong B. Khan R. R. Xiao B. Bhatta L. Vy H. M. T. . (2023). Polygenic prediction of preeclampsia and gestational hypertension. Nat. Med. 29, 1540–1549. doi: 10.1038/s41591-023-02374-9, 37248299 PMC10330886

[ref21] Howard M. C. Hoffman M. E. (2018). Variable-centered, person-centered, and person-specific approaches: where theory meets the method. Organ. Res. Methods 21, 846–876. doi: 10.1177/1094428117744021

[ref22] Jin C. H. Li R. L. Zhang L. L. Zhang Y. Li N. Wang J. H. . (2014). The revision and according validity research of China developmental scale for children. CJCHC 22, 1242–1246. doi: 10.11852/zgetbjzz2014-22-12-04

[ref23] Kyriakidou M. Chatziioannidis I. Mitsiakos G. Lampropoulou S. Pouliakis A. (2020). Neurodevelopmental outcome in extremely low birth weight infants at 2-3 years of age. Medicina (Kaunas) 56:649. doi: 10.3390/medicina56120649, 33256108 PMC7760848

[ref24] Lamont A. E. Vermunt J. K. Van Horn M. L. (2016). Regression mixture models: does modeling the covariance between independent variables and latent classes improve the results? Multivar. Behav. Res. 51, 35–52. doi: 10.1080/00273171.2015.1095063, 26881956 PMC4865372

[ref25] Li J. H. Zhao J. Z. Hua L. Hu X. L. Tang L. N. Yang T. . (2023). Efficacy of children neuropsychological and behavioral scale in screening for autism Spectrum disorders through a combination of developmental surveillance. Curr. Med. Sci. 43, 592–601. doi: 10.1007/s11596-023-2698-5, 37115393

[ref26] Lipner E. O'Brien K. J. Pike M. R. Ered A. Ellman L. M. (2023). Environmental risk factors and cognitive outcomes in psychosis: pre-, perinatal, and early life adversity. Curr. Top. Behav. Neurosci. 63, 205–240. doi: 10.1007/7854_2022_378, 35915384 PMC9892366

[ref27] Lubke G. Neale M. C. (2006). Distinguishing between latent classes and continuous factors: resolution by maximum likelihood? Multivar. Behav. Res. 41, 499–532. doi: 10.1207/s15327906mbr4104_4, 26794916

[ref28] Malacova E. Regan A. Nassar N. Raynes-Greenow C. Leonard H. Srinivasjois R. . (2018). Risk of stillbirth, preterm delivery, and fetal growth restriction following exposure in a previous birth: systematic review and meta-analysis. BJOG Int. J. Obstet. Gynaecol. 125, 183–192. doi: 10.1111/1471-0528.14906, 28856792

[ref29] Matarma T. Lagström H. Löyttyniemi E. Koski P. (2020). Motor skills of 5-year-old children: gender differences and activity and family correlates. Percept. Mot. Skills 127, 367–385. doi: 10.1177/0031512519900732, 31959076

[ref30] Miguel P. M. Pereira L. O. Silveira P. P. Meaney M. J. (2019). Early environmental influences on the development of children's brain structure and function. Dev. Med. Child Neurol. 61, 1127–1133. doi: 10.1111/dmcn.14182, 30740660

[ref31] Miranda V. P. N. Coimbra D. R. Bastos R. R. Miranda Júnior M. V. Amorim P. R. D. S. (2021). Use of latent class analysis as a method of assessing the physical activity level, sedentary behavior and nutritional habit in the adolescents' lifestyle: a scoping review. PLoS One 16:e0256069. doi: 10.1371/journal.pone.0256069, 34411143 PMC8376087

[ref32] Misirliyan S. S. Boehning A. P. Shah M. (2023). Development Milestones. Treasure Island, FL: StatPearls Publishing. 32491450

[ref33] Momany A. M. Jasper E. Markon K. E. Nikolas M. A. Ryckman K. K. (2023). Latent class analysis to characterize neonatal risk for neurodevelopmental differences. J. Child Psychol. Psychiatry 64, 100–109. doi: 10.1111/jcpp.13671, 35837724 PMC9771897

[ref34] Muthén L. K. Muthén B. O. (1998-2017). Mplus User’s Guide. Eighth Edn Los Angeles, CA: Muthén & Muthén.

[ref35] National Health Commission of the People’s Republic of China (2017). WS/T 580–2017: Developmental Milestone Checklist for Children aged 0–6 Years. Beijing: China Standard Press.

[ref36] Ng R. Chin E. Hong Y. Zhou X. Tan A. (2023). Performing pediatric neuropsychological evaluation with Chinese patients: a narrative review of the literature and recommendations for practice. Clin. Neuropsychol. 37, 930–958. doi: 10.1080/13854046.2023.2218572, 37266929

[ref37] Otieno W. A. Nyikal R. A. Mbogoh S. G. Rao E. J. O. (2023). Adoption of farm biosecurity practices among smallholder poultry farmers in Kenya - an application of latent class analysis with a multinomial logistic regression. Prev. Vet. Med. 217:105967. doi: 10.1016/j.prevetmed.2023.105967, 37406503

[ref38] Samerei S. A. Aghabayk K. Shiwakoti N. Mohammadi A. (2021). Using latent class clustering and binary logistic regression to model Australian cyclist injury severity in motor vehicle-bicycle crashes. J. Saf. Res. 79, 246–256. doi: 10.1016/j.jsr.2021.09.005, 34848005

[ref40] Silveira P. P. Portella A. K. Goldani M. Z. Barbieri M. A. (2007). Developmental origins of health and disease (DOHaD). J. Pediatr. 83, 494–504. doi: 10.2223/JPED.1728, 18074050

[ref41] Sly P. Blake T. Islam Z. (2021). Impact of prenatal and early life environmental exposures on normal human development. Paediatr. Respir. Rev. 40, 10–14. doi: 10.1016/j.prrv.2021.05.007, 34148806

[ref42] Spurk D. Hirschi A. Wang M. Valero D. Kauffeld S. (2020). Latent profile analysis: a review and “how to” guide of its application within vocational behavior research. J. Vocat. Behav. 120:103445. doi: 10.1016/j.jvb.2020.103445

[ref43] Strobel N. A. Richardson A. Shepherd C. C. J. McAuley K. E. Marriott R. Edmond K. M. . (2020). Modelling factors for aboriginal and Torres Strait islander child neurodevelopment outcomes: a latent class analysis. Paediatr. Perinat. Epidemiol. 34, 48–59. doi: 10.1111/ppe.12616, 31820463

[ref44] Tucker-Drob E. M. Briley D. A. (2014). Continuity of genetic and environmental influences on cognition across the life span: a meta-analysis of longitudinal twin and adoption studies. Psychol. Bull. 140, 949–979. doi: 10.1037/a0035893, 24611582 PMC4069230

[ref45] Vermunt J. K. (2010). Latent class modeling with covariates: two improved three-step approaches. Polit. Anal. 18, 450–469. doi: 10.1093/pan/mpq025

[ref46] Vermunt J. K. Magidson J. (2009). “Latent class cluster analysis,” in Applied Latent class Analysis, eds. Hagenaars J. McCutcheon A., vol. 11 (Cambridge: Cambridge University Press).

[ref47] Wang M. C. Bi X. Y. (2017). Advanced Topics in Latent Variable Modeling and Mplus Application. Chongqing: Chongqing University Press.

[ref39] Wang Y. N. (2024). Comparative analysis of parental care and grandparental care on children’s dynamic drawing. J. Shaanxi Norm. Univ. Preschool Educ. 34, 13–20.

[ref48] Warembourg C. Maitre L. Tamayo-Uria I. Fossati S. Roumeliotaki T. Aasvang G. M. . (2019). Early-life environmental exposures and blood pressure in children. J. Am. Coll. Cardiol. 74, 1317–1328. doi: 10.1016/j.jacc.2019.06.069, 31488269 PMC8713646

[ref49] Wei X. J. (2019). A Study on the Characteristics of Maternal and Grandmother Care for Children Aged 2–6 and their Relationship with Child Behavioral Problems. Taiyuan, China: Shanxi Medical University.

[ref50] Weller B. E. Bowen N. K. Faubert S. J. (2020). Latent class analysis: a guide to best practice. J. Black Psychol. 46, 287–311. doi: 10.1177/0095798420930932

[ref51] WHO (2012). Guideline: Use of Multiple Micronutrient Powders for Home Fortification of Foods Consumed by Infants and Children 6–23 Months of Age. Geneva: World Health Organization. 24501787

[ref53] Yamamoto N. Maruyama K. Saito I. Tomooka K. Tanigawa T. Kawamura R. . (2023). Latent profile analysis approach to the relationship between daily ambulatory activity patterns and metabolic syndrome in middle-aged and elderly Japanese individuals: the toon health study. Environ. Health Prev. Med. 28:57. doi: 10.1265/ehpm.23-00110, 37766543 PMC10569967

[ref54] Yang Y. F. (2023). Rating Scalaes for Children’s Developmental Behavior and Mental Health. 2nd Edn Beijing: People’s Medical Publishing House Co., LTD.

[ref55] Zhang H. Wang S. Tuo L. Zhai Q. Cui J. Chen D. . (2022). Relationship between maternal vitamin D levels and adverse outcomes. Nutrients 14:4230. doi: 10.3390/nu14204230, 36296914 PMC9610169

