# Supplementary material for: Latent profile analysis and influence factors of neurodevelopment in children aged 0–6 years in China
Source: Front Psychol. 2026 Jul 6;17:1705627. doi: 10.3389/fpsyg.2026.1705627 (PMC13381046; doi:10.3389/fpsyg.2026.1705627)
Supplement: Supplementary file 1 [file Supplementary_file_1.docx]

**Supplementary Materials**

**Title:** Latent Profile Analysis and Associated Factors of Neurodevelopment in Children Aged 0–6 Years in China.

**Manuscript ID:** [1705627]

**S1. Model fit indices for 1- to 5-class solutions**

Supplementary Table S1. Fit indices for latent profile models with 1–5 classes (N=2,297)

| No. of classes | AIC | BIC | aBIC | Entropy | LMR *p* | BLRT *p* | Smallest class % |
| --- | --- | --- | --- | --- | --- | --- | --- |
| 1 | 4392.131 | 4449.520 | 4417.748 | – | – | – | – |
| 2 | 2077.793 | 2169.616 | 2118.781 | 0.805 | <.001 | <.001 | 28.57% (n=656) |
| 3 | 1726.612 | 1852.869 | 1782.971 | 0.797 | <.001 | <.001 | 18.86% (n=433) |
| 4 | 1296.677 | 1457.367 | 1368.406 | 0.755 | 0.0245 | <.001 | 17.42% (n=400) |
| 5 | 1222.947 | 1418.070 | 1310.046 | 0.758 | 0.2989 | <.001 | 4.18% (n=96) |

*Note:* AIC = Akaike Information Criterion; BIC = Bayesian Information Criterion; aBIC = sample-size adjusted BIC; LMR = Lo-Mendell-Rubin test; BLRT = bootstrap likelihood ratio test. The 2‑class solution was selected due to the highest entropy, parsimony, and interpretability.

**S2. Mean percentile scores for 3-class and 4-class solutions**

Supplementary Table S2. Mean age‑standardized percentile scores across five CDSC‑II developmental domains for the 3‑class solution (N=2,297)

| Domain | Class 1 (n= 468) | Class 2 (n=433) | Class 3 (n=1396) |
| --- | --- | --- | --- |
| Gross motor | 0.545 | 0.252 | 0.594 |
| Fine motor | 0.634 | 0.254 | 0.698 |
| Language | 0.581 | 0.255 | 0.634 |
| Adaptive behavior | 0.297 | 0.228 | 0.765 |
| Social behavior | 0.501 | 0.210 | 0.663 |

Supplementary Figure S1. Percentile means for the five attributes within each latent class for Three-class solution.

**Supplementary Table S3.** Mean age‑standardized percentile scores across five CDSC‑II developmental domains for the 4‑class solution (N=2,297)

| Domain | Class 1  (n = 400 ) | Class 2  (n= 442 ) | Class 3  (n= 466 ) | Class 4  (n= 988 ) |
| --- | --- | --- | --- | --- |
| Gross motor | 0.247 | 0.569 | 0.380 | 0.670 |
| Fine motor | 0.238 | 0.661 | 0.473 | 0.774 |
| Language | 0.248 | 0.601 | 0.438 | 0.700 |
| Adaptive behavior | 0.174 | 0.286 | 0.679 | 0.794 |
| Social behavior | 0.199 | 0.511 | 0.516 | 0.705 |

Supplementary Figure S2. Percentile means for the five attributes within each latent class for Four-class solution.

**S3. Sensitivity analysis: Adjustment for child age**

**Supplementary Table S4. Results of the two-class regression mixture model adjusting for child age (months)** (N=2,297)

| Development indicators | Class 1 (Ordinary) mean | Class 2 (Excellent) mean | Predictors of Class 1 (Ordinary vs. Excellent) | OR | 95% CI | P-value |
| --- | --- | --- | --- | --- | --- | --- |
| GM (gross motor) | 0.284 | 0.608 | LBW (low birth weight) | 2.09 | 1.19, 3.66 | 0.010 |
| FM (fine motor) | 0.323 | 0.704 | PRETERM (preterm birth) | 5.23 | 3.13, 8.73 | <0.001 |
| LANG (language) | 0.298 | 0.646 | PREG_COMP (pregnancy complications) | 1.43 | 1.15, 1.77 | 0.002 |
| ADAPT (adaptive) | 0.282 | 0.673 | SOLID_FOOD (introduction of solid food) | 0.90 | 0.71, 1.13 | 0.348 |
| SOCIAL (social) | 0.264 | 0.649 | PARENTING (parenting behavior score) | 0.63 | 0.38, 1.06 | 0.081 |
| **Model fit** |  |  | AGE (child age, months) | 1.02 | 1.01, 1.03 | 0.001 |
| Loglikelihood = -946.859 |  |  | SEX (female vs. male) | 0.67 | 0.54, 0.82 | <0.001 |
| AIC = 1931.719 |  |  |  |  |  |  |
| BIC = 2040.758 |  |  |  |  |  |  |
| Entropy = 0.818 |  |  |  |  |  |  |
| **Class proportions** | 28.6% | 71.4% |  |  |  |  |

*Notes:* OR = odds ratio; CI = confidence interval. LBW, PRETERM, PREG_COMP, SOLID_FOOD, PARENTING are binary (1 = presence/higher score direction as in original scale). AGE is continuous (months). SEX: female vs. male. Class 1 is the 'ordinary development group' (28.6%) and Class 2 the 'excellent development group' (71.4%). **OR > 1 indicates higher odds of belonging to the Ordinary Development Group (Class 1).**"

**S4. Mplus syntax for age‑sensitivity analysis**

**Supplementary Appendix S1.** Mplus 8.3 syntax for the two‑class regression mixture model with child age as an additional covariate.

TITLE: 2-class regression mixture model - final model

DATA: FILE = cdsc_data.dat;

VARIABLE:

NAMES = id gm fm lang adapt social lbw preterm parenting solid_food preg_comp age sex area;

USEVARIABLES = gm fm lang adapt social lbw preterm parenting solid_food preg_comp age sex;

CLASSES = c (2);

MISSING = ALL (-999);

ANALYSIS:

TYPE = MIXTURE;

ESTIMATOR = MLR;

STARTS = 500 100;

STITERATIONS = 20;

MODEL:

%OVERALL%

! Class membership predicted by covariates (age and sex only)

c#1 ON lbw preterm preg_comp solid_food parenting age sex;

%c#1%

! Class 1: Ordinary Development Group (lower means)

[gm fm lang adapt social];

gm fm lang adapt social (1); ! Equal variances across classes

%c#2%

! Class 2: Excellent Development Group (higher means)

[gm fm lang adapt social];

gm fm lang adapt social (1);

OUTPUT: TECH1 TECH4 TECH8 TECH11 TECH14;

PLOT: TYPE = PLOT3;

SERIES = gm fm lang adapt social(*);

**S5. Additional rationale for selecting the 2‑class solution**

As discussed in the main manuscript (Section 3.2), the 2‑class solution was chosen over higher‑class solutions because:

1. **Highest entropy (0.805)** , indicating the clearest class separation.
2. The LMR test became **non‑significant for the 5‑class solution** (p=0.299) and **borderline for the 4‑class solution** (p=0.0245), suggesting over-extraction.
3. The 3‑ and 4‑class solutions split the Ordinary Development Group into subgroups that differ **quantitatively** (i.e., overall severity) rather than showing qualitatively different domain‑specific patterns (see Supplementary Tables S2–S3). All profiles are parallel.
4. The smallest class in the 5‑class solution was only **4.18% (≈96 children)** , which would compromise statistical power and replicability.

Thus, the 2‑class solution is the most parsimonious, interpretable, and replicable model for this dataset.
